# Capsule Endoscopy versus Magnetic Resonance Enterography for Evaluation of Pediatric Small Bowel Crohn’s Disease: Prospective Study

**DOI:** 10.3390/jcm11102760

**Published:** 2022-05-13

**Authors:** Jae-Yeon Hwang, Sang-Wook Moon, Yeoun Joo Lee, Jae Hong Park, Yong-Woo Kim, Tae Un Kim, Hwaseong Ryu

**Affiliations:** 1 Department of Radiology, Research Institute for Convergence of Biomedical Science and Technology, Pusan National University Yangsan Hospital, College of Medicine, Pusan National University, Yangsan 50612, Korea; jyhwang79@gmail.com (J.-Y.H.); kyw47914@gmail.com (Y.-W.K.); kimtaeun78@hanmail.net (T.U.K.); yocomeon83@naver.com (H.R.); 2 Department of Pediatrics, Research Institute for Convergence of Biomedical Science and Technology, Pusan National University Yangsan Hospital, College of Medicine, Pusan National University, Yangsan 50612, Korea; soulwalker@naver.com (S.-W.M.); jjhongpark@pusan.ac.kr (J.H.P.)

**Keywords:** small bowel Crohn’s disease, child, pediatric, magnetic resonance enterography, capsule endoscopy

## Abstract

Background: Magnetic resonance enterography (MRE) and capsule endoscopy (CE) are currently used for the evaluation of small bowel involvement in pediatric Crohn’s disease (CD). Several studies have been conducted to investigate the usefulness and diagnostic accuracy of each test. However, only a few studies have been conducted to compare the performance of both tests in the assessment of pediatric small bowel CD upon diagnosis and during follow-up. Therefore, the purpose of this study was to assess the diagnostic accuracy and diagnostic consistency of CE and MRE for the evaluation of pediatric small bowel CD at the time of diagnosis and during follow-up. Methods: Fifteen patients with pediatric CD were recruited for this study. They underwent MRE and CE concomitantly at the time of diagnosis and 10–12 weeks and one year after induction therapy for CD. MRE was interpreted using MRE global score (MEGS) and bowel wall inflammation severity diffusion-weighted imaging score (BWI-DWIS), whereas CE was interpreted using Lewis’s score (LS). The two diagnostic modalities were then compared. Results: Eleven patients completed MRE and CE at the time of diagnosis. Analysis of the results showed that LS had a strong correlation with MEGS and BIS-DWIS (ρ = 0.633, *p* = 0.037, and ρ = 0.629, *p* = 0.038, respectively). Nine patients completed three MREs and three CEs. LS significantly decreased throughout the sessions (*p* = 0.044), whereas MEGS and BIS-DWIS did not show any statistically significant changes. When LS was compared with MEGS and BIS-DWIS, both MRE indicators showed statistically significant differences throughout the sessions. Conclusions: At the time of diagnosis, the severity indexes of MRE and CE showed very good agreement. However, throughout management, MRE and CE did not show consistent changes.

## 1. Introduction

The current treatment target for Crohn’s disease (CD) is achieving mucosal healing, which is well documented in the Selecting Therapeutic Targets in Inflammatory Bowel Disease (STRIDE) strategy [1]. However, transmural healing is another necessary treatment goal. Hence, mucosal and transmural healing should be facilitated by the thorough evaluation of clinical and laboratory data, endoscopy, or imaging for the initial diagnosis and follow-up.

The involvement of the small bowel in the development of CD is relatively more difficult to determine than that of the upper gastrointestinal tract and colon. Various imaging techniques are used for this purpose. Currently, even laboratory tests are performed more actively to evaluate the involvement of the small bowel in CD than before. Thus, CD apparently has a higher prevalence now than it did traditionally.

Capsule endoscopy (CE) is performed to visualize the small intestinal mucosa in patients with CD. However, children suspected with CD cannot undergo CE since they experience difficulties in swallowing capsules. CE is a passive imaging technique that cannot be manipulated. Its disease detection rates have been reported as 50–70% because of the challenges in changing the direction of the camera or washing the bubbles. Moreover, a risk of capsule retention and sudden stenosis remains for CE.

Computed tomography enterography (CTE) and magnetic resonance enterography (MRE) have been developed as imaging modalities used to visualize the wall thickness of the entire bowel, its lumen and mucosa, and extra-enteric manifestations. However, CTE is unsuitable for pediatric patients because of the risk of ionizing radiation. Meanwhile, MRE is considered potentially advantageous since it lacks the use of radiation, provides dynamic phase artifacts, and offers good soft-tissue contrast [2]. Regarding the balance between diagnostic accuracy and radiation concerns, CTE is preferred as the initial imaging method as it provides superior spatial resolution, whereas MRE is recommended as a recurrent imaging method and used extensively for patients with CD.

Several investigators explored the diagnostic yields (DY) of CE and MRE in pediatric CD [2,3,4,5]. These studies showed that CE and MRE have similar diagnostic performances. However, CE showed a superior DY in detecting proximal small bowel disease [4,5] than MRE. Nevertheless, the studies comparing the diagnostic performance of CE and MRE for small bowel CD in pediatric patients during the follow-up period are few. Therefore, this study aimed to assess the diagnostic accuracy and consistency of CE and MRE in the assessment of pediatric CD at the initial diagnosis and during the follow-up period.

## 2. Methods

### 2.1. Ethical Statements

This study was conducted after approval was obtained from the institutional review board of Pusan National University Yangsan Hospital (04-2015-022). Written informed consent was provided voluntarily by all participating patients and their legal guardians after they were provided with sufficient information regarding the purpose and method of the study. All methods were performed following the relevant guidelines and regulations.

### 2.2. Subjects

Between December 2015 and July 2017, we planned to enroll 15 patients in this study. The inclusion criteria were as follows: age between 10 and 18 years, patients diagnosed with Crohn’s disease endoscopically and histologically, clinically suspected with active small bowel CD, and were candidates for induction treatment for pediatric CD. The exclusion criteria were as follows: having a contraindication for intravenous administration of gadolinium-based contrast media; having no tolerance for the oral administration of contrast media; having a contraindication for CE; requiring sedation for MRE; and having limitations of holding the breath for 10–15 s. For each patient, MRE and CE were conducted before induction therapy was initiated (first session), after 10–12 weeks (second session), and after one year (third session).

### 2.3. Study Techniques

#### 2.3.1. MRE Technique

MRE was performed after oral administration of 1000–1500 mL of 0.1% *w*/*v* Barium Sulfate solution (Easymark, Taejoon Pharmaceuticals, Seoul, Korea) or Polyethylene Glycol solution. MRE was performed using a 3-T MR scanner (Skyra, Siemens Medical Solutions, Erlangen, Germany). The following sequences were performed: coronal T2-weighted half-Fourier single-shot turbo spin (HASTE) echo sequences with and without fat suppression; axial T2-weighted HASTE with fat suppression; coronal and axial T2-like steady-state gradient-echo sequences with fat suppression; coronal free-breathing diffusion-weighted imaging (DWI) with b-value of 50 and 800 s/mm^2^ and apparent diffusion coefficient mapping; dynamic coronal T1-weighted spoiled gradient echo sequences with fat suppression, including pre-contrast scan and enteric phase and portovenous phase scans after intravenous administration of 0.2 mL/kg bodyweight of a gadolinium-based contrast medium (Dotarem, Guerbet, Roissy, France) at 2 mL/s followed by a saline flush; and axial post-contrast T1-weighted spoiled gradient echo sequences with fat suppression. Before MRE and CE, 5 mg Cimetropium Bromide (Algiron, Boehringer Ingelheim, Ingelheim, Germany) was administered intravenously to minimize the urge for peristalsis. Detailed scan parameters are presented in Table A1.

#### 2.3.2. CE Technique

CE was performed on the same day or the following day for patients without stenosis after MRE. CE was performed without any further preparation, except instructing the patients to ingest nothing by mouth after undergoing MRE. Clear water ingestion was permitted during the procedure. In the case of performing colonofibroscopy together, bowel preparation was performed according to the colonoscopy. PillCamTM SB-II capsule was used for the evaluation, which was self-administered orally to the patients without any endoscopic assistance. All images were reviewed using a video production software (RAPID ver. 8, Given Imaging, Yokneam Illit, Israel).

### 2.4. Interpretation of MRE and CE Images

#### 2.4.1. Interpretation of MRE Images

Before analysis, the MRE images were re-ordered randomly after patient identifiers were removed, and were interpreted using a dedicated PACS workstation. The images were interpreted by two radiologists; one with ten years of experience as a board-certified pediatric radiologist and the other with four years of experience as a board-certified gastrointestinal radiologist. Decisions were reached in consensus. Since the consensus reading reflected our clinical practice and this study was a preliminary exploration of the monitoring of small bowel CD in pediatric patients, we chose the single consensus reading approach for this study.

Interpretation of the MRE findings was analyzed by dividing the small bowel into the proximal, middle-to-distal, and terminal ileal segments. The terminal ileum was defined as the distal 30 cm segment of the ileum. Quantification of the disease severity in each bowel segment was assessed by calculating the MRE global score (MEGS) [6]. We modified the MEGS to reflect the length of the diseased segment for the segmental scores: the multiplication score per segment, which is determined by the length of the diseased segment, was multiplied by the segmental bowel wall scores. The total score was calculated as follows: score per segment + additional score per patient (lymphadenopathy + comb sign + abscess + fistula).

The DWI score (BIS-DWIS) was calculated by adding the DWIS to the segmental MEGS, which indicates the severity of the bowel wall inflammation. The signal intensity of the bowel wall on DWI images was scored from 0 to 3 [7]. In addition, the total bowel inflammation severity score was defined as the summation of the BIS-DWIS of three segments. The formulas used to calculate the MEGS and BIS-DWIS are described in Table A2. Image quality was assessed by measuring the motion artifacts, degree of bowel distention, and the overall image quality of coronal post-contrast T1-weighted images. The assessment form for image quality is provided in Table A3.

#### 2.4.2. Interpretation of Capsule Endoscopy Images

CE images were reviewed by two pediatric gastroenterologists who had two years and ten years of experience in CE interpretations. Their agreement data was accepted. Images were reviewed at a rate of fewer than 15 frames per second. Visualization quality was assessed before interpretation. An image was rated as excellent if there was >75% visibility of the small bowel. If the visibility of small bowel was 50–75%, the image was considered as inadequately illuminated; visibility of small bowel < 50% was apparently caused by inadequate bowel preparation.

The small bowel transit time was calculated for each segment of the small bowel. The three segments according to the transit time were the proximal, middle-to-distal, and terminal ileum based on the small bowel transit time. For the assessment of disease activity in CD, a known CE scoring system, the Lewis score (LS), was used [8,9]. This is an index calculated by evaluating the presence of villous edema, ulcer, and stenosis in each segment. A score between 135 and 790 is considered mild, whereas scores ≥ 790 are considered moderate-to-severe. The scores were collected for each test, and scores for each segment were separately collected.

### 2.5. Statistical Analysis

The patients’ demographic and descriptive data for MRE and CE scores are presented as mean ± standard deviation or median scale and interquartile range for continuous variables and as percentages for categorical variables. Student’s *t*-test or Mann–Whitney test were used to assess continuous variables, whereas the χ^2^ test or Fisher’s exact test were used for categorical variables. Spearman’s rank correlation coefficient (ρ) was calculated to assess the correlation between LS and MEGS and that between LS and BIS-DWIS. Spearman’s rank correlation coefficient values were interpreted as follows: ρ < 0.1 indicated no correlation; ρ values in the range 0.1–0.3 indicated weak to modest correlation; ρ values ranging 0.3–0.49 indicated moderate correlation; ρ values ranging 0.5–0.79 indicated strong correlation; and ρ ≥ 0.8 indicated a moderately strong correlation. Time-dependent differences were analyzed for patients who completed all three sessions of MRE and CE by repeated measures ANOVA. Two MRE scales (MEGS and BIS-DWIS) and LS for CE were compared between segments and the total score using repeated-measures analysis of variance. A *p*-value ≤ 0.05 was considered statistically significant.

## 3. Results

### 3.1. Patients’ Characteristics

Fifteen patients were enrolled in this study. One patient withdrew consent prior to participation. Two patients with intestinal stenosis in the first MRE were excluded from further examination with CE. One patient without a small intestine lesion during the first MRE was excluded from further examination with CE. Two patients withdrew consent voluntarily after the second MRE and CE session because they expressed difficulty in undergoing MRE and CE due to poor compliance. Overall, 12 patients underwent the first MRE, 11 patients underwent the first CE, 11 patients underwent the second MRE and CE sessions, and nine patients completed all three sessions of MRE and CE. The mean age of the 11 patients who underwent at least two examination sessions was 14.3 ± 2.0 (median, 14.6; range: 10.3–16.9) years old. Six of the patients were boys. The median Pediatric CD Activity Index (PCDAI) recorded at the first session was 27.5 (range, 20.0–47.5), and the median erythrocyte sedimentation rate (ESR) at the first session was 28.0 (range, 11–74) mm/h. The median PCDAI at the second session was 5.0 (range, 0.0–30.0), and the median ESR at second session was 14.0 (range, 2–81) mm/h. The median PCDAI at the third session (n = 9) was 15.0 (range, 0.0–32.5), and the median ESR at the third session was 16.0 (range: 2–64) mm/h. The treatment of patients before each session is described in Table 1.

### 3.2. Magnetic Resonance Enterography

**Figure 1:**
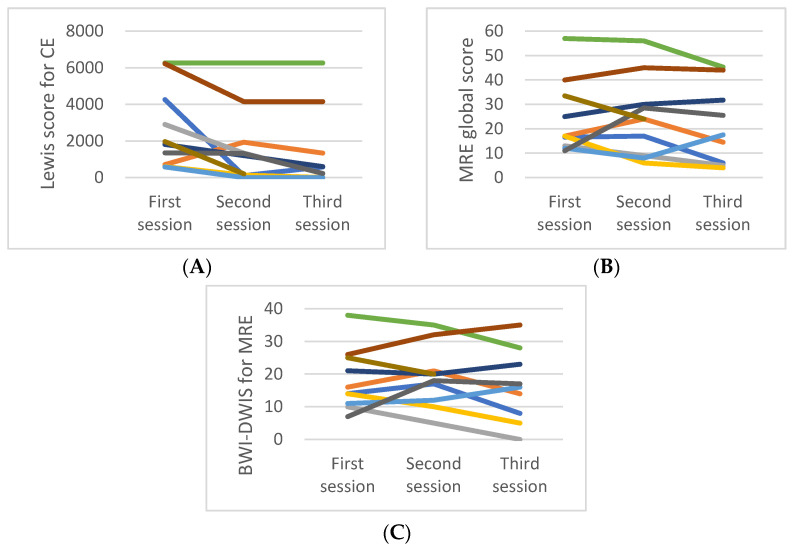
Changes of Lewis score of capsule endoscopy (CE) (**A**), magnetic resonance enterography (MRE) global score (**B**), and bowel wall inflammation severity diffusion-weighted image score (BWI-DWIS) for MRE (**C**) in 11 pediatric Crohn’s disease patients. The same color in each picture represents the same patient.

The segmental score, total MEGS, and BIS-DWIS are summarized in Table 1. The changes in the MEGS and BIS-DWIS of each patient are shown in Figure 1. The global MEGS and BIS-DWIS for the 11 MRE examinations performed in the first session were 17 (IQR; 12.5–29.5) and 14 (IQR: 9.5–23), respectively; those for the 10 MRE examinations performed in the second session were 24 (IQR: 9–30) and 19 (IQR: 12–21), respectively; and those for the nine MRE examinations performed in the third session were 18 (IQR: 5.75–35) and 16 (IQR: 7.25–24.25), respectively (Table 2). Lymphadenopathy of >1 cm was observed in 20 MRE images, whereas enteric fistula was observed in three. None of the MRE images showed abscess formation.

Regarding image quality, the median score of the motion artifacts was 4 (IQR: 3–5). Three MRE images were deemed to have moderate to severe artifacts. The median score of bowel distension was 4 (IQR: 3–4), and three MRE images showed suboptimal bowel distention. The median score of overall image quality was 4 (IQR: 3–4). Three MRE images had suboptimal image quality.

### 3.3. Capsule Endoscopy

Among the 31 CE examinations performed for 11 patients, 26 (83.9%) showed excellent visualization quality, two had inadequate illumination, and the three cases with poor visualization quality were attributed to inadequate bowel preparation. Of the three CE examinations with poor visualization quality, one in the second session was excluded from the CE scoring due to marked insufficient visibility. The mean stomach transition time was 43 min, and the mean small bowel transition time was 345 ± 151 min. Two CE examinations performed for one patient were completed in the terminal ileum. The capsule did not pass through the colon for more than 14 h.

Regarding LS, eight patients had moderate to severe scores and three patients showed mild scores in the 11 CE examinations performed in the first session (Table 1). The median LS for the first CE examination performed for 11 patients was 1808 (IQR: 600–4260), that for the second CE examination performed for 10 patients was 1256 (IQR: 129–2490), and that for the third CE examination performed for nine patients was 580 (IQR: 117–2744). The changes in the LS of each patient are shown in Figure 1.

For the 11 CE examinations performed in the first session, the median LS of the first, second, and third segments of the small intestine was 278 (IQR; 225–1968), 450 (IQR; 225–1808), and 712 (IQR; 225–1808), respectively. These results reflect that the terminal ileum had the highest LS (Table 3).

### 3.4. Comparison of Magnetic Resonance Enterography and Capsule Endoscopy

A strong correlation was found between the LS and the MEGS and the LS and the BIS-DWIS of the 11 patients examined in first session (ρ = 0.633, *p* = 0.037 and ρ = 0.629, *p* = 0.038, respectively). The LS and the MEGS and the LS and the BIS-DWIS of the 10 patients examined in the second session also showed a strong correlation (ρ = 0.736, *p* = 0.015 and ρ = 0.669, *p* = 0.035, respectively). The LS and the MEGS of the nine patients examined in the third session showed strong correlation as well (ρ = 0.669, *p* = 0.049). However, their LS and BIS-DWIS did not show any statistically significant correlation.

Time-dependent differences were analyzed for the nine patients who completed three sessions of MRE and CE. LS significantly decreased during the course of the sessions, while MEGS and BIS-DWIS did not show any statistically significant changes (Table 4). When LS was compared with MEGS and BIS-DWIS, both MRE indicators showed statistically significant differences from LS over the course of the sessions (Table 5). The time-dependent differences between involved segments showed no statistical significance.

## 4. Discussion

In this study, we assessed the diagnostic accuracy and consistency of CE and MRE for the evaluation of pediatric CD. We conducted this study to evaluate and compare disease severity using CE and MRE at the time of initial diagnosis and during the follow-up process of the disease. We found a correlation between the disease severity scoring of MRE and CE in each of the sessions performed. However, when all three sessions were compared, we confirmed that the change in the CE and MRE disease severity scores was statistically different. Regarding CE, in which mucosal ulcer status is evaluated first, 66% of patients were considered to be in remission or mild disease at one year after diagnosis. On the contrary, MRE showed no significant change at the time of diagnosis, at 10–12 weeks, and at one year. We hypothesized that this discordance between CE and MRE in monitoring small bowel CD was because CE evaluates the severity index of lesions based only on the mucosal change, whereas MRE assesses not only mucosal changes, but transmural involvement as well.

Transmural healing is a very important treatment goal. However, the STRIDE strategy [1], which presents the treatment target for CD, focuses on mucosal healing as a therapeutic goal. Therefore, clinicians and researchers should carefully judge whether MRE is an appropriate method for confirming treatment response to determine if the treatment plan needs changes. There are several studies that compared MRE and CE for the treatment response of CD. A prospective study of 48 adults with ileal CD showed that MRE could demonstrate the ulcer healing with 90% accuracy and endoscopic remission with 83% accuracy versus the observation of only mucosal ulcers using ileo-colonoscopy and MRE before treatment and at week 12 of treatment [10]. Elżbieta Krzesiek et al. reported transmural healing in 16.7% of patients and improvement in 55.5% examinations when MRE was used as a follow-up modality to assess children. They concluded that MRE is a useful, tolerable, and non-invasive procedure that can assess the treatment response [11]. However, to our knowledge, this study is the first to compare MRE and CE directly to confirm the treatment response in small bowel CD.

We used the LS to measure the severity of CD assessed using CE. LS quantifies the evaluation of the extent of disease invasion in the small intestine of patients with CD. By evaluating edema at the villi level, as well as the size of the ulcer, early changes in CD and active disease are recognized simultaneously, and a high addition point is given to stricture, the advanced disease status. In addition, the length of the invaded segment is evaluated to determine disease activity in small bowel CD. Therefore, LS is used in CD studies to study disease severity [9,12].

The simple endoscopic score for CD (SES-CD) is a known indicator of the disease severity of CD as observed using CE. SES-CD is a colonoscopy evaluation index that evaluates the size of an ulcer and the area of invasion. It was developed as a colonoscopy index and has been used as a similar concept for evaluation of the small intestine. Therefore, SES-CD is a simple index for evaluating gross lesions that is used in CE studies, especially to score lesions in the terminal ileum [13,14]. Moreover, SES-CD scoring is used to evaluate the cross-sectional size and area of ulcers.

There are several MRE-based indices used for the quantification of inflammation in CD, including Magnetic Resonance Index of Activity (MaRIA), MEGS, Clermont score, Crohn disease MR index (CDMI), and Lemann scoring system [15]. Although the MaRIA score is the most investigated index and has been proven to have suitable performance in many studies [13,14,15,16], it is used to assess ileocolic CD, and it incorporates fewer MRE parameters than other scoring systems. In the present study, we used MEGS and BIS-DWIS to evaluate the entire small bowel. In addition, the MEGS system is based on the CDMI, which incorporates a wide spectrum of MRE parameters for both mural and extramural disease manifestations, including bowel wall enhancement, wall thickness, ulcers, mural and peri-mural T2 signal changes, lymphadenopathy, comb sign, abscess, and fistula. It uses a simple grading system for each MRE parameter, and the total score is obtained by multiplying the score per segment by score per segment and adding additional scores per patient [6,15].

DWI is an MR technique that expresses the diffusion of water molecules in biological tissues. Although the exact mechanism underlying the restricted diffusion noted in the active inflammation of the bowel in CD is not clear, restricted diffusion is a non-specific sign of mural inflammation in CD and is a complementary and supportive finding [17]. A recently published meta-analysis showed that DWI has heterogeneous diagnostic accuracy depending on the study design, blinding to the intravenous contrast-enhanced MRE, and reference standards. The summary sensitivity of DWI was 84% and the specificity was 73% when endoscopy and surgical pathology were considered reference standards [18]. A recent study showed that DWI MRE was noninferior to contrast-enhanced MRE for evaluating small bowel CD [7].

MRE and CE as clinical diagnostic modalities for small bowel CD may be randomly selected by the clinician according to the personal preference, ease of result interpretation, and the patients’ preferences as well. The difference between the two diagnostic methods is theoretically explained. However, only a few studies discuss the similarities and differences existing in their practical use. Some studies investigated the differences and commonalities of MRE and CE. Marina et al. [4] compared three diagnostic modalities of MRE, small intestinal contrast ultrasonography, and CE for evaluating small intestinal lesions in pediatric CD. They reported similar degrees of diagnostic performance for each modality. However, only the diagnostic performance of each examination technique for lesion assessment in each segment of the small bowel was assessed while not evaluating the correlation between diagnostic performance and disease severity. Jordi et al. [13] set CE as a reference standard and compared the three MRE-based indices (MaRIA, Clermont, and London indices) and reported that all three MRE scores showed high diagnostic accuracy for the assessment of disease activity in small bowel CD in adult patients, and that the MaRIA index had the best operational characteristics. The diagnostic accuracies of MRE indices have been compared in some studies, whereas the disease activity has been compared in other studies according to various reading methods. In a meta-analysis comprising 10 clinical studies that compared MRE and CE, both test methods showed similar DYs. However, CE showed a relatively higher DY in proximal small bowel CD [19]. In this study, the 11 MRE and CE examinations conducted in the first session showed a high disease severity agreement. However, due to the small number of patients enrolled, it was not possible to obtain information on the DY of each technique for the small intestine (proximal, middle-to-distal, terminal ileum).

Per previous reports, CE is preferred over MRE for small bowel assessments in adult patients. One study reported that during bowel preparation and examination, discomfort was reported with a significantly higher frequency for MRE than for CE. Whereas Lahat et al. reported that 78% patients preferred CE over MRE [20]. Although patient discomfort was not investigated in this study, we noted that patients who dropped out had difficulty with undergoing MRE. Two patients dropped out because they expressed discomfort with the bowel preparation process and long scanning time for MRE. The results of MRE showed a very high agreement with those of CE at the initial examination. However, in the first year of follow-up, there was no significant change compared to CE. MRE is very useful as an initial diagnostic modality for small intestinal CD in pediatric patients. However, considering the difficulties associated with the test and the difference between its disease severity scoring and that of CE during follow-up, careful attention should be paid to the use of the MRE as a follow-up examination method in the first year of disease treatment for childhood CD.

## 5. Limitations

There were several limitations in this study. First, both MRE and CE are very difficult to perform in a child and, therefore, the tests tend not to be performed excellently. For CE, the visualization quality of three examinations indicated inadequate bowel preparation, whereas one was hardly interpretable. Second, only nine patients (60.0%) were able to participate in the entire study, leading to low statistical power. In addition, in this study, patient treatment was not controlled, so the patient’s treatment policy was not consistent. This may have influenced the interpretation of the test. Nevertheless, this is the first study in which the difference between two diagnostic methods used for the assessment of small intestine involvement in pediatric CD was recognized by comparing the findings at the time of diagnosis and at multiple follow-up time points.

## 6. Conclusions

Clinicians who treat pediatric CD should understand the characteristics and limitations of MRE and CE and their individual advantages before using them to determine the treatment plan. In the initial diagnosis of CD, MRE and CE show appreciable agreement. However, if only MRE or CE test can be performed during the follow-up period, there are limitations to the results provided by each technique. Therefore, it is necessary to recognize the advantages and disadvantages of these test methods. In addition, clinical symptoms, laboratory tests, etc. should be comprehensively evaluated to determine the best treatment policy to be used.

## Figures and Tables

**Table 1:** Treatment of patients before each session, CE preperation and test performed together.

		First Session (n = 11)	Second Session (n = 10)	Third Session (n = 9)
Prior treatment	No (diagnosis)	9		
	CS		7	
	EEN + IM		1	
	IM			5
	Biologics	2		2
	Biologics + IM			1
	Biologics + IM + CS		2	1
Preperation before CE	NPO with clear liquid	7	10	6
	Polyethylene glycol	2		1
	Sodium picosulfate/magnesium citrate	2		2
Test performed together	MRE only	6	10	7
	MRE + DFS	1		
	MRE + DFS + CFS	2		1
	MRE + CFS	2		1

CS, corticosteroid; EEN, exclusive enteral nutrition; IM, immunomodulator; CE, capsule endoscopy; MRE, MR enterography; DFS, duodenofibroscopy; CFS, colonofibroscopy.

**Table 2:** Summary of scores obtained by magnetic resonance enterography (MRE) and capsule endoscopy (CE).

		First Session (n = 11)	Second Session (n = 10)	Third Session (n = 9)
Proximal SB	LS score for CE	251.5 (225–1284)	416 (168.5–1354)	8.5 (6.5–10.5)
	MEGS for MRE	1.0 (0–5)	1.5 (0–4)	3.0 (0–5.75)
	BIS-DWIS for MRE	1.5 (0–5.5)	3.0 (2–5)	8.5 (6.5–10.5)
Middle to distal SB	LS score for CE	225 (0–1012)	135 (0–1336)	11.5 (6–14)
	MEGS for MRE	2.0 (2–8)	4.5 (2–6)	2.0 (0–10)
	BIS-DWIS for MRE	2.0 (0–5)	5.5 (2–6)	11.5 (6–14)
Terminal ileum	LS score for CE	225 (0–402.75)	135 (0–585)	10 (7.25–11.5)
	MEGS for MRE	9.0 (6.5–13)	10 (5.5–14)	10 (5.5–14)
	BIS-DWIS for MRE	3.0 (0–4.5)	3.0 (0–7.25)	10 (7.25–11.5)
Total	LS score for CE	1579 (589–3852)	1256 (135–1936)	580 (170.75–2040)
	MEGS for MRE	17 (12.5–29.5)	24 (9–30)	18 (5.75–35)
	BIS-DWIS for MRE	14 (9.5–23)	19 (12–21)	16 (7.25–24.25)

Numbers represent median values. Numbers in parentheses represent interquartile ranges. SB, small bowel; MEGS, magnetic resonance enterography global score; BWI-DWIS, bowel wall inflammation severity diffusion-weighted imaging scores; LS, Lewis score.

**Table 3:** Changes of lesion severity according to the Lewis score for Capsule Endoscopy.

Lewis Score	First Session (n = 11)	Second Session (n = 10)	Thrid Session (n = 9)
Remission (<135)	0	2	2
Mild disease (135–790)	3	2	4
Moderate to severe disease (≥790)	8	6	3

**Table 4:** Time-dependent differences according to the sessions on Lewis score for CE, MEGS for MRE and BIS-DWIS for MRE on 9 patients.

	First Session	Second Session	Third Session	*p*-Value
Lewis score for CE	2744.89 (773.60)	1827.44 (698.67)	1487.78 (737.28)	0.044 *
MEGS for MRE	23.22 (5.17)	24.89 (5.74)	21.67 (5.35)	0.535
BIS-DWIS for MRE	17.44 (3.21)	18.89 (3.25)	16.22 (3.73)	0.459

* *p*-value < 0.05. CE, capsule endoscopy; MEGS, magnetic resonance enterography global score; MRE, magnetic resonance enterography; BWI-DWIS, bowel wall inflammation severity diffusion-weighted imaging scores.

**Table 5:** Time-dependent differences according to the sessions between Lewis score for CE and two MRE scores of MEGS and BIS-DWIS on 9 patients.

CE			MRE				
First Session	Second Session	Third Session	First Session	Second Session	Third Session	F-Score	*p*-Value
2744.89 (773.60)	1827.44 (698.67)	1487.78 (737.28)	MEGS				
			23.22 (5.17)	24.89 (5.74)	21.67 (5.35)	5.11	0.0192 *
			BIS-DWIS				
			17.44 (3.21)	18.89 (3.25)	16.22 (3.73)	5.11	0.0193 *

* *p*-value < 0.05. CE, capsule endoscopy; MEGS, magnetic resonance enterography global score; MRE, magnetic resonance enterography; BWI-DWIS, bowel wall inflammation severity diffusion-weighted imaging scores.

## Data Availability

The datasets analyzed during the current study are not publicly available due to ethical reasons but are available from the corresponding author on reasonable request.

## Appendix A

**Table A1:** Scan parameters of MRE.

	T2-Weighted Half-Fourler Sequence		T2-Weighted Half-Fourier Sequence	T2-Like Steady-State Gradient-Echo Sequence	DWI Sequence	T1-Weighted CE Sequence ^†^	
Plane	Coronal	Coronal	Axial	Coronal	Coronal	Coronal	Axial
Fat saturation	Yes	No	Yes	Yes	Yes	Yes	Yes
Breath hold	Yes	Yes	Yes	Yes	No	Yes	Yes
Repetition time (ms)/echo time (ms)/flip angle (degrees)	1000/126/148	1000/126/148	968/123/155	3.8/1.6/63	5200/55/90	5.8/2.5/9	5.9/2.5/9
Field of view (mm)	360 ×360	360 × 360	300 × 260	330 × 430	300 × 430	380 × 380	300 × 300
Matrix	320 × 288	320 × 256	320 × 211	320 × 256	148 × 118	384 × 250	384 × 246
Section thickness/gap (mm)	5.0/5.5	5.0/5.5	5.0/5.0	5.0/5.0		2.0/2.0	2.0/2.0
Parallel imaging factor	3	3	2	2	4	3	3
Echo planar imaging factor					118		
Number of averages	1	1	1	1	3	1	1
b factor (s/mm^2^)					50, 800		

^†^ Volumetric T1-weighted spoiled gradient echo sequences. Bolus trigger technique was used for determining scan delay. Acquisition of enteric phase scan was started in 3 s after visualization of the contrast media in both iliac vessels and the acquisition of portovenous phase scan was started approximately in 60 s after administration of the contrast media.

**Table A2:** MR Enterography scores for severity of bowel wall inflammation.

	Score 0	Score 1	Score 2	Score 3
Mural thickness	<3 mm	3–5 mm	5–7 mm	>7 mm
Mural T2 signal	Equivalent to normal bowel wall	Dark grey on fat-suppressed images	Light grey on fat-suppressed images	White high signal similar to that of luminal content
Perimural T2 signal	Equivalent to normal mesentery	Increase in mesentery signal but no fluid	Small fluid rim (<2 mm)	Large fluid rim (≥2 mm)
T1 enhancement	Equivalent to normal bowel wall	Increased bowel wall signal but significantly less than nearby vascular structures	Increased bowel wall signal but somewhat less than nearby vascular structures	Bowel wall signal similar that of nearby vascular structures
Mural enhancement pattern	N/A or homogeneous	Mucosal	Layered	
DWI signal intensity	No increased diffusion restriction	Increased DWI signal but slightly less than that of lymph nodes	Increased DWI signal similar to that of lymph node	Increased DWI signal higher than that of lymph node
Length of disease segment	×1.0: 0–5 cm ×1.5: 5–15 cm ×2: >15 cm			
Additional score per segment	Lymph node (>1 cm): score 5 Comb sign, abscess, fistula: score 1			

**Table A3:** Assessment form of image quality scores.

Motion Artifact	
1	Uninterpretable
2	moderate to severe artifacts resulting in markedly decreased diagnostic confidence
3	moderate artifacts resulting in moderately decreased diagnostic confidence
4	minimal artifacts not affecting diagnostic confidence
5	no artifacts with excellent image quality
Bowel distention	
1	luminal collapse compromising diagnostic interpretation
2	markedly suboptimal bowel distention resulting in markedly decreased diagnostic confidence
3	moderately suboptimal distention resulting in moderately decreased diagnostic confidence
4	good but suboptimal bowel distention not affecting diagnostic confidence
5	excellent optimal bowel distention
Overall image quality	
1	uninterpretable
2	markedly decreased diagnostic confidence due to suboptimal image quality
3	moderately decreased diagnostic confidence because of suboptimal image quality
4	mild degradation in image quality not affecting diagnostic confidence
5	, excellent image quality with high diagnostic confidence
